# Clinical and sociodemographic determinants of older breast cancer survivors’ reports of receiving advice about exercise

**DOI:** 10.1007/s10549-024-07460-1

**Published:** 2024-09-30

**Authors:** Kaitlyn M. Wojcik, Oliver W. A. Wilson, Dalya Kamil, Padma Sheila Rajagopal, Mara A. Schonberg, Jinani Jayasekera

**Affiliations:** 1 Intramural Research Program, Health Equity and Decision Sciences Research Laboratory, National Institute on Minority Health and Health Disparities, National Institutes of Health, Bethesda, MD 20892 USA; 2 Center for Cancer Research at the National Cancer Institute, National Institutes of Health, Bethesda, MD USA; 3 Division of General Medicine, Department of Medicine, Beth Israel Deaconess Medical Center, Boston, MA USA

**Keywords:** Breast cancer survivors, Exercise advice, Healthcare providers, Survivorship care

## Abstract

**Purpose:**

Exercise offers various clinical benefits to older breast cancer survivors. However, studies report that healthcare providers may not regularly discuss exercise with their patients. We evaluated clinical and sociodemographic determinants of receiving advice about exercise from healthcare providers among older breast cancer survivors (aged ≥65 years).

**Methods:**

We used data from the Surveillance, Epidemiology, and End Results cancer registries linked to the Medicare Health Outcomes Survey (MHOS) from 2008 to 2015. We included female breast cancer survivors, aged ≥65 years, who completed the MHOS survey ≥2 years after a breast cancer diagnosis in a modified Poisson regression to identify clinical and sociodemographic determinants of reportedly receiving advice about exercise from healthcare providers.

**Results:**

The sample included 1,836 breast cancer survivors. The median age of the sample was 76 years (range: 72–81). Overall, 10.7% of the survivors were non-Hispanic Black, 10.1% were Hispanic, and 69.3% were non-Hispanic White. Only 52.3% reported receiving advice about exercise from a healthcare provider. Higher body mass index (BMI) and comorbid medical history that included diabetes, cardiovascular, or musculoskeletal disease were each associated with a higher likelihood of receiving exercise advice. Lower education levels, lower BMI, and never having been married were each associated with a lower likelihood of receiving exercise advice.

**Conclusions:**

Nearly half of breast cancer survivors aged ≥65 years did not report receiving exercise advice from a healthcare provider, suggesting interventions are needed to improve exercise counseling between providers and survivors, especially with women with lower educational attainment who have never been married.

**Supplementary Information:**

The online version contains supplementary material available at 10.1007/s10549-024-07460-1.

## Introduction

Due to improved treatment regimens [1], breast cancer mortality rates in the U.S. have declined annually by 1.3% in the past decade [2]. Currently, there are close to four million female breast cancer survivors in the U.S., the majority (67%) of whom are over the age of 65 years [3, 4]. The most recent exercise guidelines for cancer survivors recommend individualized exercise prescriptions in order to improve cancer-related health outcomes, including anxiety, depressive symptoms, physical functioning, and health-related quality of life [5]. The American Cancer Society, the American College of Sports Medicine, and the American Institute for Cancer Research recommend the equivalent of ≥150–300 min/week of moderate intensity aerobic activity (e.g., walking, swimming, cycling), along with ≥2 days/week of muscle-strengthening exercise (e.g., lifting weights) [5–10]. Despite this, a recent analysis using the National Health Interview Survey indicated that less than half of breast cancer survivors in the U.S. (36%) currently meet aerobic exercise guidelines [11]. This proportion decreases with rising age due to corresponding increases in comorbidities, intimidation of starting exercise, and lack of transportation, parks and recreation space, and guidance on exercise [12]. Other factors relevant to the general population, such as body mass index (BMI), comorbidities, or marital status, also influence survivors’ participation in physical activity [13, 14].

The American Society of Clinical Oncology recommends that all cancer survivors receive exercise counseling [15]. Previous studies have shown that healthcare provider discussions about exercise with their patients can help increase exercise participation [16]. Most cancer survivors also report wanting to discuss exercise and cardiovascular health with their clinicians during survivorship care planning [17–21]. However, despite guidelines and evidence supporting the benefits of exercise, older cancer survivors continue to report limited to no discussions about exercise from their healthcare providers in clinical settings [22–25].

There is limited data to date on individual clinical and sociodemographic characteristics of breast cancer survivors specifically that may influence how or why healthcare providers engage in conversations about exercise in clinical settings. This information could potentially help healthcare systems and providers better consistently identify, engage, and support women who may be less likely to receive advice about exercise during survivorship care planning, while also reducing potential physician-related biases. In this population-based study, we aimed to identify clinical and sociodemographic characteristics associated with the likelihood of receiving advice about exercise from healthcare providers among older breast cancer survivors. Additionally, we evaluated the comorbid health conditions associated with reported receipt of exercise advice.

## Methods

### Data source and study population

We used the National Cancer Institute’s Surveillance, Epidemiology and End Results (SEER) cancer registry data and the Centers for Medicare & Medicaid Services’ Medicare Health Outcomes Survey (MHOS), which is available within one data resource (SEER-MHOS). SEER is a national cancer surveillance network of 16 regional cancer registries covering approximately 28% of the U.S. population that collects information on cancer diagnosis, site, and histology, initial cancer therapies, and demographic characteristics [26]. We included all SEER participating sites in these analyses, which included Connecticut, Georgia, Hawaii, Idaho, Iowa, Kentucky, Louisiana, Massachusetts, New Jersey, New Mexico, New York, Utah, Greater California/Los Angeles/Greater Bay Area/San Francisco/Oakland/San Jose/Monterey, Atlanta/Greater Georgia/Rural Georgia, Seattle/Puget Sound, and Detroit [26]. MHOS is a patient-reported survey of a random sample of enrollees in Medicare Advantage that assesses health-related quality of life and evaluates performance of the plans [27].

**Fig. 1:**
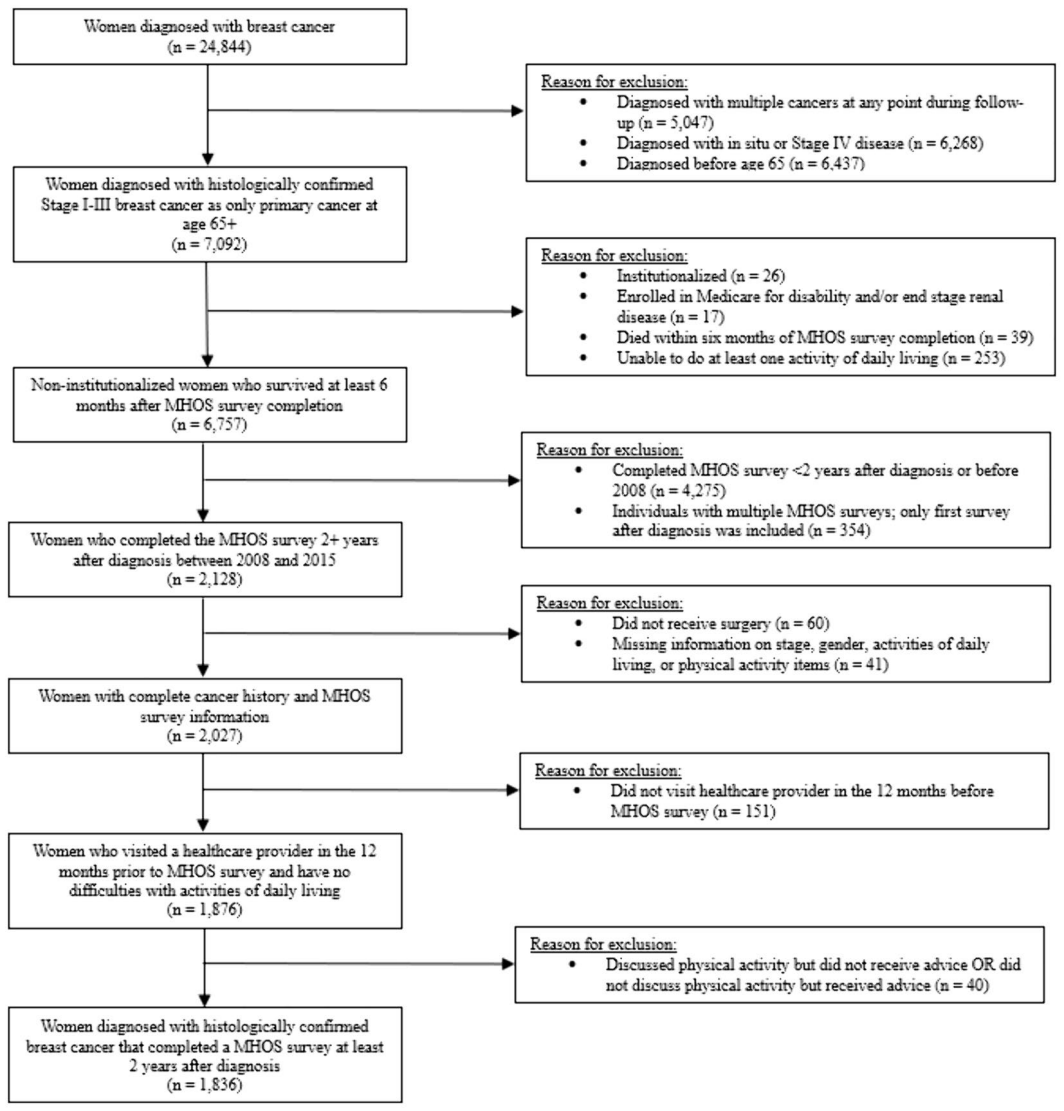
Inclusion and exclusion criteria

Consistent with previous literature, we included women diagnosed with histologically confirmed invasive breast cancer aged ≥65 years at diagnosis from SEER, and breast cancer survivors who had completed a MHOS survey at least two years after diagnosis [28–30]. We excluded participants who had missing data for reported levels of exercise and whose breast cancer diagnosis occurred at autopsy. In 2008, the U.S. national physical activity guidelines highlighted that healthcare providers should assess, counsel, and advise patients on exercise and how to do it safely [31]. Exercise guidelines for cancer survivors were introduced in 2010, which recommended two sessions per week of strength training and ≥150 min/week of aerobic activity [32]. Since physical activity guidelines for healthcare providers caring for this patient population were first published in 2008, we restricted our sample to include surveys completed from 2008 to 2015. The full list of inclusion and exclusion criteria can be found in Fig. 1. The final sample consisted of 1836 breast cancer survivors.

### Exercise advice from healthcare providers

The outcome of interest in this study was reporting receiving exercise advice from a healthcare provider. The MHOS exercise advice item stated, “In the past 12 months, did a doctor or other health provider advise you to start, increase, or maintain your level of exercise or physical activity?” Respondents were asked to either answer ‘yes’ or ‘no’ [33].

### Demographic characteristics

Based on previous studies [29, 34], we considered the following important covariates for reporting receipt of exercise counseling, including self-reported race and ethnicity (Hispanic, non-Hispanic Asian or Pacific Islander/American Indian/Alaska Native, non-Hispanic Black, and non-Hispanic White), marital status (married, divorced/separated/widowed, never married), household income (less than $20,000, $20,000–$39,999, $40,000–$79,999, $80,000 +), education level (less than high school, high school graduate or general education development (GED), some college/college graduate or more), and continuous age at survey in the analyses.

### Breast cancer history

SEER summary stage (localized, regional), receipt of radiation therapy (yes, no), receipt of surgery (partial mastectomy, lumpectomy, excision of biopsy, modified radical mastectomy), tumor grade (I, II, III, IV), estrogen receptor status, progesterone receptor status, human epidermal growth factor receptor 2 (HER2) status, and age at diagnosis were included in the analyses.

### Clinical characteristics

Based on expert opinion and a previous analysis of SEER-MHOS data [29], we also included information on other self-reported clinical characteristics, such as a woman’s history of falls in the last 12 months (yes, no), difficulty performing activities of daily living (yes, no) (includes bathing, dressing, eating, getting in/out of a chair, walking, using the toilet), BMI category (underweight: BMI <20 kg/m^2^; normal: BMI ≥20–24.9 kg/m^2^; overweight: BMI ≥25–29.9 kg/m^2^; obese/morbidly obese: BMI ≥30 kg/m^2^), general health status (excellent, very good, good, fair, poor), and comorbidity status (yes, no), including hypertension, cardiovascular disease (coronary artery disease, congestive heart failure, myocardial infarction, other heart conditions), pulmonary disease (emphysema, asthma, chronic obstructive pulmonary disease), diabetes, and musculoskeletal disease (arthritis of hip/knee or hand/wrist, osteoporosis, sciatica).

### Study design and data analysis

Descriptive statistics were used to summarize sociodemographic characteristics, cancer history, and other clinical variables. We used a modified Poisson regression model with a least absolute shrinkage and selection operator (LASSO) to identify variables associated with the likelihood of receiving advice on exercise. LASSO was used to select variables to include in the final model, prevent overfitting of models, and improve model accuracy [35]. The overall model included difficulty performing activities of daily living, race/ethnicity, marital status, education, BMI category, fall history, receipt of radiation therapy, age at diagnosis, comorbidities (yes/no), tumor grade, and time between survey and diagnosis. We also ran five separate models for each comorbidity type (hypertension, cardiovascular disease, diabetes, pulmonary disease, or musculoskeletal disease) to reduce collinearity. We tested interactions between race/ethnicity, BMI, and education. We also ran additional exploratory subgroup analyses stratified by race and ethnicity (non-Hispanic White, non-Hispanic Black, Hispanic). Statistical significance was set at *p* < 0.05. All analyses were conducted in RStudio, version 2023.06.1, and Stata, version 18.0 [36, 37]. This study was approved by the National Institutes of Health Institutional Review Board and was considered as exempt research based on use of de-identified pre-existing data.

## Results

The sample included 1,836 women diagnosed with breast cancer. The median age of the sample at the time of the survey was 76 years (range: 72–81); 69.3% were non-Hispanic White, 43.4% had a college degree or higher, 38.8% were currently married, and 32.9% had a household income of less than $20,000 per year. Most women had at least one comorbidity (93.5%), with the three most commonly reported comorbidities being musculoskeletal disease (73.7%), hypertension (69.3%), and cardiovascular disease (28.3%); 37.3% reported difficulty performing an activity of daily living. The majority of women were diagnosed with breast cancer between ages 65–74 years (68.9%), and most women were diagnosed with localized invasive breast cancer (78.4%) and had received radiation (56.6%) (Table 1).

**Table 1:** Characteristics of older breast cancer survivors in the overall sample and subgroups stratified by the receipt of exercise advice from a healthcare provider

Characteristics	Overall Sample		Has your doctor given you exercise advice?			
	N	%	Yes (n = 960)		No (n = 876)	
			n	Col%^1^	n	Col%^1^
Clinical characteristics						
Age at diagnosis (Years)						
65–69	777	42.3	429	44.7	348	39.7
70–74	488	26.6	262	27.3	226	25.8
75–79	335	18.2	161	16.8	174	19.9
≥80	236	12.9	108	11.2	128	14.6
Difficulty performing ≥1 activity of daily living						
Yes	684	37.3	382	39.8	302	34.5
No	1152	62.7	578	60.2	574	65.5
Difficulty bathing						
Yes	234	12.7	131	13.6	103	11.8
No	1602	87.3	829	86.4	773	88.2
Difficulty dressing						
Yes	147	8.0	88	9.2	59	6.7
No	1689	92.0	872	90.8	817	93.3
Difficulty eating						
Yes	59	3.2	36	3.8	23	2.6
No	1777	96.8	924	96.2	853	97.4
Difficulty getting in/out of a chair						
Yes	405	22.1	232	24.2	173	19.7
No	1431	77.9	728	75.8	703	80.3
Difficulty walking						
Yes	592	32.2	326	34.0	266	30.4
No	1244	67.8	634	66.0	610	69.6
Difficulty using the toilet						
Yes	104	5.7	61	6.4	43	4.9
No	1732	94.3	899	93.6	833	95.1
Fall history in past 12 months						
Yes	452	24.6	240	25.0	212	24.2
No/Missing	1384	75.3	720	75.0	664	75.8
BMI category						
Underweight (<18.5 kg/m^2^)	91	5.0	29	3.1	62	7.5
Normal (≥18.5- <25 kg/m^2^)	571	31.1	268	29.1	303	36.5
Overweight (≥25- < 30kg/m^2^)	558	30.4	309	33.6	249	30.0
Obese/morbidly obese (≥30 kg/m^2^)	531	28.9	315	34.2	216	26.0
General health status						
Excellent	88	4.8	43	4.6	45	5.2
Very good	436	23.7	222	23.6	214	24.7
Good	774	42.2	410	43.6	364	42.1
Fair	436	23.7	229	24.3	207	23.9
Poor	72	3.9	37	3.9	35	4.0
Comorbidities						
≥1 Comorbidity	1717	93.5	912	95.0	805	91.9
None	119	6.5	48	5.0	71	8.1
Hypertension						
Yes	1273	69.3	692	72.1	581	66.3
No/Missing	560	30.7	268	27.9	295	33.7
Cardiovascular disease						
Yes	519	28.3	301	32.1	218	25.8
No	1264	68.8	638	67.9	626	74.2
Pulmonary disease						
Yes	311	16.9	170	17.9	141	16.4
No	1499	81.6	780	82.1	719	83.6
Diabetes						
Yes	502	27.3	305	32.2	197	22.8
No/Missing	1311	71.4	643	67.8	668	77.2
Musculoskeletal disease						
Yes	1354	73.7	749	78.0	605	69.1
No/Missing	482	26.3	211	22.0	271	30.9
Tumor grade						
Grade I	526	30.1	284	30.8	242	29.3
Grade II	806	46.2	429	46.6	377	45.7
Grade III/IV	414	22.5	208	21.7	206	23.5
Missing	90	4.9	39	4.1	51	5.8
SEER summary stage						
Localized	1440	78.4	752	78.3	688	78.5
Regional	396	21.6	208	21.7	188	45.3
Radiation						
Yes	1039	56.6	560	58.3	479	54.7
No/Missing	797	43.4	400	41.7	397	45.3
Surgery						
Partial mastectomy	213	11.6	111	11.6	102	11.6
Lumpectomy	760	41.4	403	42.0	357	40.8
Excision of biopsy	265	14.4	141	14.7	124	14.2
Total mastectomy	259	14.1	131	13.6	128	14.6
Modified radical mastectomy	339	18.5	174	18.1	165	18.8
Estrogen receptor status						
Positive	1516	86.7	805	88.0	711	85.4
Negative	232	13.3	110	12.0	122	14.6
Progesterone receptor status						
Positive	1288	74.5	681	75.0	607	74.0
Negative	440	25.5	227	25.0	213	26.0
HER2 receptor status						
Positive	43	7.9	19	6.7	24	9.1
Negative	504	92.1	263	93.3	241	90.9
Age at survey (Median [IQR])	76 [72, 81]		76 [72, 81]		77 [73, 82]	
Time between survey and diagnosis (median [IQR])	4.6 [3.1, 6.5]		4.4 [3.1, 6.4]		4.7 [3.0, 6.6]	
Sociodemographic characteristics						
Race/ethnicity						
Hispanic	185	10.1	110	11.5	75	8.6
Non-Hispanic American Indian/Alaska Native/Asian or Pacific Islander	175	10.0	98	10.2	77	8.8
Non-Hispanic Black	197	10.7	100	10.4	97	11.1
Non-Hispanic White	1272	69.3	650	67.8	622	71.4
Marital status						
Married	713	38.8	391	41.6	322	37.7
Divorced/separated/widowed	992	54.0	513	54.5	479	56.2
Never married	89	4.8	37	3.9	52	6.1
Education level						
Less than high school	370	20.2	176	18.9	194	22.9
High school graduate or GED	616	33.6	309	33.1	307	36.2
Some college/college degree	796	43.4	448	48.0	348	41.0
Household income						
< $20,000	604	32.9	317	40.7	287	43.4
$20,000–$39,999	448	24.4	241	30.9	207	31.3
$40,000–$79,999	283	15.4	158	20.3	125	18.9
≥$80,000	106	5.8	63	8.1	43	6.5

*BMI* Body mass index, *HER2* human epidermal growth factor 2, *GED* general education development, *IQR* interquartile range

^1^Column percentage calculated (cell number/column total) × 100

E.g., Col. % for no difficulties in performing activities of daily living among those who received advice about exercise from a doctor (578/960) × 100 = 60.2%

The multivariable modified Poisson regression analysis showed that, from a sociodemographic standpoint, the strongest associated variables were education and marital status. Compared to breast cancer survivors with a college degree or more, those with less than a high school education or those with a high school degree or a GED had a 23% (Relative Risk (RR): 0.77; 95% confidence interval (CI): 0.67–0.88), and 12% (RR: 0.88; 95% CI: 0.80–0.98) lower likelihood of receiving exercise advice from a healthcare provider, respectively. Breast cancer survivors who were never married had a 21% lower likelihood of receiving exercise advice than those who were married (RR: 0.79; 95% CI: 0.61–1.03), and these results were trending towards significance. From a clinical standpoint, the strongest associated variables were BMI and age. With every one-year increase in age, survivors had a 1% lower likelihood of receiving exercise advice (RR: 0.99; 95% CI: 0.98–1.00); these results were trending towards significance. Compared to those with normal BMI (≥18.5–<25 kg/m^2^), breast cancer survivors who were underweight (<18.5 kg/m^2^) reported a 26% lower likelihood (RR: 0.74; 95% CI: 0.54–1.03) of receiving advice, while breast cancer survivors who were overweight (≥25–<30 kg/m^2^) and obese (≥30 kg/m^2^) reported a 21% (RR: 1.21; 95% CI: 1.08–1.36) and 26% (RR: 1.26; 95% CI: 1.11–1.42) higher likelihood of receiving advice, respectively. Survivors with at least one comorbidity had a 29% higher likelihood of receiving advice compared to those without any comorbidities (RR: 1.29; 95% CI: 1.03–1.62). Compared to non-Hispanic White breast cancer survivors, non-Hispanic Asian or Pacific Islander/American Indian/Alaska Native survivors had a 17% higher likelihood of receiving exercise advice (RR: 1.17; 95% CI 1.01–1.36) (Table 2). Difficulty performing ≥ 1 activity of daily living, fall history, receipt of radiation, and tumor grade were not associated with receipt of exercise advice from a healthcare provider. The interactions between race/ethnicity, BMI, and education were trending towards significance. However, there was inadequate sample size to conduct subgroup analyses.

**Table 2:** Factors associated with the receipt of exercise advice from healthcare providers in a multivariable modified poisson regression analysis^*^

Variable	Category	Relative risk	95% Confidence interval	
			Lower	Upper
Clinical characteristics				
BMI	Normal (≥18.5–<25 kg/m^2^)	Reference		
	Underweight (<18.5 kg/m^2^)	0.74	0.54	1.03
	Overweight (≥25–<30 kg/m^2^)	1.21	1.08	1.36
	Obese/Morbidly Obese (≥30 kg/m^2^)	1.26	1.11	1.42
Comorbidities	≥1 Comorbidity	1.29	1.03	1.62
	None	Reference		
Difficulty performing ≥ 1 activity of daily living	Yes	1.09	0.99	1.21
	No	Reference		
Fall history in past 12 months	Yes	1.04	0.94	1.16
	No	Reference		
Radiation	Yes	1.07	0.98	1.18
	No	Reference		
Grade	Grade I	Reference		
	Grade II	0.97	0.88	1.08
	Grade III	0.92	0.81	1.05
	Grade IV	0.46	0.08	2.54
Age at diagnosis	Continuous	0.99	0.98	1.00
Time from survey to diagnosis	Continuous	0.99	0.98	1.01
Sociodemographic characteristics				
Race/ethnicity	Non-Hispanic White	Reference		
	Non-Hispanic American Indian/Alaska Native/Asian or Pacific Islander	1.17	1.01	1.36
	Non-Hispanic Black	0.98	0.83	1.14
	Hispanic	1.14	0.99	1.32
Marital status	Married	Reference		
	Divorced/separated/widowed	1.02	0.93	1.13
	Never married	0.79	0.61	1.03
Education	College degree or more	Reference		
	High school graduate or GED	0.88	0.80	0.98
	Less than high school	0.77	0.67	0.88

^*^Represents a fully adjusted model; all variables in table were included in the model

*BMI* Body mass index, *GED* general education development

The results for exploratory subgroup analyses stratified by race and ethnicity are provided in Supplemental Table 1. Breast cancer survivors with less than a high school education were less likely to receive exercise advice from a healthcare provider across non-Hispanic White (RR: 0.71; 95% CI: 0.58–0.86) and Hispanic (RR: 0.61; 95% CI: 0.45–0.83) groups.

The comorbidity-specific models showed that breast cancer survivors with musculoskeletal disease (RR: 1.22; 95% CI: 1.08–1.37), diabetes (RR: 1.15; 95% CI: 1.04–1.27) and cardiovascular disease (RR: 1.11; 95% CI: 1.01–1.22) showed a higher likelihood of receiving exercise advice. Hypertension and pulmonary disease were not associated with reports of receiving exercise advice (Table 3; Supplemental Tables 2, 3, 4, 5, 6).

**Table 3:** Individual comorbidities associated with the receipt of exercise advice from healthcare providers in multivariable modified poisson regression analyses

Comorbidities	Relative risk	95% Confidence interval	
		Lower	Upper
Hypertension	1.07	0.96	1.20
Cardiovascular disease	1.10	1.01	1.22
Pulmonary disease	1.01	0.90	1.14
Diabetes	1.15	1.04	1.27
Musculoskeletal disease	1.22	1.08	1.37

Each row represents a separate model; adjusted for difficulty performing activities of daily living, race/ethnicity, marital status, education, body mass index, fall history, radiation, tumor grade, age at diagnosis, time from survey to diagnosis, and any other comorbidities

## Discussion

To our knowledge, this is the first study that assesses sociodemographic and contextual determinants of receiving exercise advice from healthcare providers solely among breast cancer survivors. Breast cancer survivors have unique needs relating to exercise; for instance, due to adverse effects of treatment, preexisting conditions, and lower exercise participation following diagnosis, women with breast cancer may have a higher risk of cardiovascular disease-related mortality than the general population [38, 39]. Additionally, breast cancer survivors may face reduced muscular strength and mobility due to treatment-related side effects [40, 41]. Therefore, it is important that clinicians discuss the benefits and potential risks of exercise with these women.

Our results show that only about half of the older breast cancer survivors (52.3%) had received exercise advice from their healthcare providers. This is consistent with a previous study [29] using the SEER-MHOS dataset (2008–2014), which found that only about half (53.4%) of older breast, colorectal, and prostate cancer survivors reported discussing exercise with their healthcare providers. Previous studies not specific to the cancer survivor population have also shown that, among adult patients, only about half (50.2%) of healthcare providers counsel their patients on either diet, exercise, or weight control [34, 42–44]. Healthcare providers cite numerous reasons for limited engagement in discussions about exercise in clinical practice, including the lack of time and clinical tools to support personalized discussions [22–24, 45]. Providers, especially those in primary care, report feeling time pressure during their visits with patients [46], and often there is not enough time for them to discuss exercise. Providers also report feeling like they have a lack of expertise and knowledge about exercise [47, 48]. Survey studies have shown that clinicians may not be aware of the current exercise guidelines, as it remains absent from the formal education of many healthcare professions [47, 48]. Additionally, providers may not be aware of the different types, frequency, and duration of exercise that a patient may need based on the patient’s risk of cardiovascular disease and other comorbidities [49, 50]. Healthcare providers who offer advice to cancer survivors may focus on discussing the benefits of exercise for physical and functional health gains rather than discussing the full range of benefits that exercise may offer breast cancer survivors, including improvements in quality of life during treatment [19]. Thus, it is important that clinicians are provided training and education about exercise, information on referral programs, and training on how to discuss exercise effectively in different patient populations.

We found that sociodemographic and contextual factors in this population are associated with the likelihood of receiving advice about exercise from a healthcare provider. Overall, lower levels of education were associated with a lower likelihood of receiving advice about exercise from a healthcare provider. A similar relationship was observed in non-Hispanic White and Hispanic women; however, these subgroup analyses were considered exploratory due to limited sample size. While we observed a strong relationship between lower levels of education and lower likelihood of being advised about exercise, potential reasons for this are unclear. Educational attainment has been shown to be a significant predictor of pre-existing exercise levels, with lower levels of education associated with less exercise [51]. This relationship could be driven by lower exercise self-efficacy, low income stability, and limited social support associated with lower levels of education [52]. It is possible that patients may have prompted this discussion among providers. It may be important to develop targeted interventions to increase discussions and exercise participation among these individuals since lower levels of education are associated with other concurrent, intersecting challenges to survivorship, including obesity, morbidity, and mortality [51]. Additionally, providers should consider a patients’ level of health literacy when delivering exercise advice; information should be given to patients at a level that they can understand in order to increase their self-efficacy, enabling them to feel confident about exercising [53, 54].

We also observed that survivors who had never been married were significantly less likely to report receiving exercise advice than those who had been married. However, studies show that married women report higher levels of exercise and leisure time activity compared to their single counterparts [13]. This may be explained by the marriage protection theory, which suggests that marriage provides health benefits due to increased social and financial support [55]. It is essential for providers to consider encouraging survivors who have never been married to also pursue exercise, especially among those who may not have access to other avenues of social support, given the potential benefits of marriage.

Among clinical factors, BMI had the strongest association with receiving exercise advice among breast cancer survivors. Older breast cancer survivors who had overweight or obesity showed a higher likelihood of receiving exercise advice. While there may certainly be additional benefits to exercise relevant to these populations, this can also potentially feed into pre-existing biases by providers that alienate patients of greater weight and worsen health outcomes if advice is not delivered effectively, while not adequately serving patients of normal weight who may benefit from greater muscle mass/functional status. Additionally, compared to normal weight, underweight survivors had a lower likelihood of receiving exercise advice. Underweight older survivors may be nearing death, so it might be inappropriate for clinicians to recommend exercise to these individuals [56].

With advancing age, breast cancer survivors were less likely to receive exercise advice from their healthcare providers, even when considering comorbidities. This is particularly concerning given that older women participate in the least exercise [57]. There could be inadvertent bias against older patients, where providers may have negative ageist stereotypes that may lead to poorer quality of care [58]. Providers need to encourage older breast cancer survivors to exercise, and future interventions are needed to ensure that providers deliver advice effectively to this patient population. Providers may consider offering exercise advice while considering different modalities, such as home-based exercise, that may increase exercise among this patient population [59].

Diabetes, cardiovascular, and musculoskeletal diseases were also associated with a higher likelihood of receiving exercise advice from a healthcare provider. However, in our study, pulmonary disease and hypertension were not associated with the receipt of exercise advice. Interestingly, prior studies have shown that exercise significantly reduces morbidity and mortality related to all of these conditions [60–64], encouraging the possibility of providers to expand the patients with comorbidities whom they encourage to exercise.

The intersection of cultural factors with the factors that we measured may influence breast cancer survivors’ ability to exercise, and these factors should be considered when providers engage in discussions about exercise with their patients. For example, in our study, we found that non-Hispanic American Indian/Alaska Native/Asian or Pacific Islander breast cancer survivors had a greater likelihood of receiving exercise advice. These findings are encouraging considering the lower levels of exercise [65–68] and higher rates of obesity and comorbidities reported in these groups [69]. Previous studies have shown that American Indian and Alaska Native women were more likely to exercise if they had higher levels of social support [70, 71]. However, most existing exercise interventions have failed to incorporate community-based strategies to help these women rely on their social environments [71]. Similarly, Asian American women report cultural reasons, lack of time due to keeping their traditions, and feeling like exercise was not appropriate for women as reasons for not exercising [72]. Community-based and culturally relevant exercise interventions that incorporate social and cultural norms, values, and beliefs may help engage these women in discussions about exercise. Interventions that are designed with community members, aligned with their values, delivered at a relevant health literacy level, and address specific barriers relevant to the community are more effective and can help increase the efficacy of exercise programs [73–75].

Our study presents several limitations. Due to the small sample size, while we could analyze single factors, we were unable to run subgroup analyses stratified by multiple factors (e.g., race and education; BMI and comorbidities). Additionally, the sample sizes for non-Hispanic Asian, Pacific Islander, American Indian, and Alaska Native breast cancer survivors were small. As a result, we were unable to conduct separate analyses for these women. There is a critical need to increase the inclusion of these underrepresented groups in registry data. As we focused on women aged over 65 years who may be clinically underserved with regard to exercise advice, our findings may not be generalizable to younger women. Our sample also only included women with Medicare Advantage plans, and patients who are on other types of health insurance may demonstrate different rates of receiving exercise advice from providers based on alternative factors (such as income). For instance, we were not able to evaluate the impact of the potential differences in physician services and payment structures in Medicare Advantage and fee-for-service Medicare plans that may influence exercise discussions [76, 77]. The SEER-MHOS dataset does not collect follow-up data on the uptake of exercise; therefore, we were unable to assess whether exercise discussions/advice with a healthcare provider resulted in a behavioral change or maintenance. Additionally, the SEER-MHOS dataset provided a single binary variable for receiving exercise advice from a clinician. Future research may include variables that describe the characteristics of exercise advice including ‘what’, ‘when’, ‘how’, and ‘why’ exercise advice was provided to the patient. Additionally, we were not able to include chemotherapy in our analyses due to the lack of completeness of the chemotherapy variable reported in SEER [78]. Also, we only used data from 2008 to 2015; as a result we were unable to evaluate the impact of the COVID-19 pandemic on the receipt of exercise advice from healthcare providers. As the data becomes available, future research may consider evaluating the impact of the COVID-19 pandemic on the receipt of exercise advice among older adults. Finally, given the nature of the SEER-MHOS questionnaire, our results may have been influenced by recall bias, where women who were already engaging in exercise were more likely to report that their clinicians recommended exercise. However, the intersection of older age and exercise patterns among breast cancer survivors remains a space with limited data.

In summary, our study provides important data to help healthcare providers identify, engage, and provide advice about exercise to older breast cancer survivors. Our findings also indicate that exercise prescriptions may require consideration of both individual clinical and sociodemographic factors. A clinical decision tool or a conversation aid specific to breast cancer survivors, extending the capabilities and level of individualization offered by existing tools such as ‘The Exercise and Screening for You’ tool [79], could potentially help address barriers to communicating the benefits of exercise in diverse breast cancer settings and could potentially help improve the overall quality of breast cancer survivorship care.

## Supplementary Information

Below is the link to the electronic supplementary material.

Supplementary file1 (DOCX 47 KB)

## Data Availability

The SEER-MHOS datasets analyzed during the current study are not publicly available due to the risk of re-identification. The data are available to investigators for research purposes, but approval is required to obtain the data.
