# Supplementary material for: Clinical and sociodemographic determinants of older breast cancer survivors’ reports of receiving advice about exercise
Source: Breast Cancer Res Treat. 2024 Sep 30;208(3):643–55. doi: 10.1007/s10549-024-07460-1 (PMC11522097; doi:10.1007/s10549-024-07460-1)
Supplement: Supplementary file 1 — Supplementary file1 (DOCX 47 KB) [file 10549_2024_7460_MOESM1_ESM.docx]

Supplemental Table 1. Modified Poisson Regression Results – Subgroup Analysis by Race and Ethnicity

| **Variable** | **Category** | **Non-Hispanic White**  **(n = 1,272)** | | | **Non-Hispanic Black**  **(n = 197)** | | | **Hispanic**  **(n = 185)** | | |
| --- | --- | --- | --- | --- | --- | --- | --- | --- | --- | --- |
|  |  | **Relative Risk** | **95% Confidence Interval** | | **Relative Risk** | **95% Confidence Interval** | | **Relative Risk** | **95% Confidence Interval** | |
|  |  |  | **Lower** | **Upper** |  | **Lower** | **Upper** |  | **Lower** | **Upper** |
| **Clinical Characteristics** | | | | | | | | | | |
| **BMI** | Normal (≥18.5-<25kg/m^2^) | Reference | | | | | | | | |
|  | Underweight (<18.5kg/m^2^) | 0.85 | 0.60 | 1.21 | * | * | * | * | * | * |
|  | Overweight (≥25-<30kg/m^2^) | 1.27 | 1.09 | 1.46 | 0.99 | 0.64 | 1.52 | 1.13 | 0.76 | 1.70 |
|  | Obese/Morbidly Obese (≥30kg/m^2^) | 1.29 | 1.11 | 1.50 | 1.26 | 0.85 | 1.87 | 1.31 | 0.86 | 2.00 |
| **Comorbidities** | None | Reference | | | | | | | | |
|  | Any | 1.30 | 0.99 | 1.69 | 0.97 | 0.33 | 2.89 | 1.73 | 0.80 | 3.72 |
| **Difficulty Performing at Least 1 Activity of Daily Living** | No | Reference | | | | | | | | |
|  | Yes | 1.06 | 0.93 | 1.20 | 1.37 | 1.01 | 1.85 | 0.93 | 0.68 | 1.26 |
| **Fall History in Past 12 Months** | No | Reference | | | | | | | | |
|  | Yes | 1.10 | 0.97 | 1.25 | 0.85 | 0.56 | 1.28 | 0.92 | 0.67 | 1.26 |
| **Radiation** | No | Reference | | | | | | | | |
|  | Yes | 1.03 | 0.92 | 1.16 | 1.04 | 0.78 | 1.38 | 1.40 | 1.06 | 1.85 |
| **Grade** | Grade I | Reference | | | | | | | | |
|  | Grade II | 0.97 | 0.86 | 1.11 | 1.10 | 0.77 | 1.58 | 0.85 | 0.64 | 1.12 |
|  | Grade III | 0.95 | 0.82 | 1.11 | 0.84 | 0.55 | 1.32 | 0.70 | 0.47 | 1.03 |
|  | Grade IV | 0.60 | 0.12 | 3.07 | - | - | - | - | - | - |
| **Age at Diagnosis** | Continuous | 0.99 | 0.98 | 1.00 | 0.99 | 0.96 | 1.02 | 0.99 | 0.97 | 1.02 |
| **Time from Survey to Diagnosis** | Continuous | 0.99 | 0.97 | 1.02 | 1.02 | 0.96 | 1.09 | 1.01 | 0.95 | 1.07 |
| **Sociodemographic Characteristics** | | | | | | | | | | |
| **Marital Status** | Married | Reference | | | | | | | | |
|  | Divorced/Separated/Widowed | 1.02 | 0.91 | 1.14 | 0.85 | 0.60 | 1.19 | 0.93 | 0.72 | 1.20 |
|  | Never married | 0.76 | 0.54 | 1.07 | 0.65 | 0.32 | 1.29 | 0.86 | 0.42 | 1.76 |
| **Education** | College degree or more | Reference | | | | | | | | |
|  | High school graduate or GED | 0.89 | 0.79 | 1.01 | 0.87 | 0.60 | 1.26 | 0.77 | 0.57 | 1.03 |
|  | Less than high school | 0.71 | 0.58 | 0.86 | 1.00 | 0.69 | 1.44 | 0.61 | 0.45 | 0.83 |

BMI: body mass index; HS: high school; OR: odds ratio; CI: confidence interval; GED: general education development; * = Sample size too small, so included in reference category

Supplemental Table 2. Modified Poisson Regression Results – Hypertension*

| **Variable** | **Category** | **Relative Risk** | | **95% Confidence Interval** | | |
| --- | --- | --- | --- | --- | --- | --- |
|  |  |  |  | **Lower** | | **Upper** |
| **Clinical Characteristics** | | | | | | |
| **BMI** | Normal (≥18.5-<25kg/m^2^) | Reference | | | | |
|  | Underweight (<18.5kg/m^2^) | 0.74 | | 0.53 | | 1.02 |
|  | Overweight (≥25-<30kg/m^2^) | 1.20 | | 1.06 | | 1.35 |
|  | Obese/Morbidly Obese (≥30kg/m^2^) | 1.22 | | 1.08 | | 1.39 |
| **Hypertension** | No | Reference | | | | |
|  | Yes | 1.07 | | 0.96 | | 1.20 |
| **Any Other Comorbidities** | None | Reference | | | | |
|  | Any | 1.35 | | 1.15 | | 1.58 |
| **Difficulty Performing at Least 1 Activity of Daily Living** | No | Reference | | | | |
|  | Yes | 1.07 | 0.97 | | 1.18 | |
| **Fall History in Past 12 Months** | No | Reference | | | | |
|  | Yes | 1.04 | | 0.94 | | 1.16 |
| **Radiation** | No | Reference | | | | |
|  | Yes | 1.08 | | 0.98 | | 1.18 |
| **Grade** | Grade I | Reference | | | | |
|  | Grade II | 0.96 | | 0.87 | | 1.07 |
|  | Grade III | 0.92 | | 0.81 | | 1.04 |
|  | Grade IV | 0.48 | | 0.09 | | 2.48 |
| **Age at Diagnosis** | Continuous | 0.99 | | 0.98 | | 1.00 |
| **Time from Survey to Diagnosis** | Continuous | 0.99 | | 0.97 | | 1.01 |
| **Sociodemographic Characteristics** | | | | | | |
| **Race/Ethnicity** | Non-Hispanic White | Reference | | | | |
|  | Non-Hispanic American Indian/Alaska Native/Asian or Pacific Islander | 1.16 | | 1.00 | | 1.35 |
|  | Non-Hispanic Black | 0.99 | | 0.84 | | 1.15 |
|  | Hispanic | 1.15 | | 1.00 | | 1.33 |
| **Marital Status** | Married | Reference | | | | |
|  | Divorced/Separated/Widowed | 1.00 | | 0.91 | | 1.10 |
|  | Never married | 0.78 | | 0.60 | | 1.00 |
| **Education** | College degree or more | Reference | | | | |
|  | High school graduate or GED | 0.89 | | 0.81 | | 0.99 |
|  | Less than high school | 0.77 | | 0.67 | | 0.88 |

BMI: body mass index; OR: odds ratio; CI: confidence interval; GED: general education development

*Represents a fully adjusted model; all variables in table were included in the model. The total number of breast cancer survivors included in this analysis is 1,583.

Supplemental Table 3. Modified Poisson Regression Results – Cardiovascular Disease*

| **Variable** | **Category** | **Relative Risk** | | **95% Confidence Interval** | | |
| --- | --- | --- | --- | --- | --- | --- |
|  |  |  |  | **Lower** | | **Upper** |
| **Clinical Characteristics** | | | | | | |
| **BMI** | Normal (≥18.5-<25kg/m^2^) | Reference | | | | |
|  | Underweight (<18.5kg/m^2^) | 0.73 | | 0.52 | | 1.02 |
|  | Overweight (≥25-<30kg/m^2^) | 1.23 | | 1.09 | | 1.39 |
|  | Obese/Morbidly Obese (≥30kg/m^2^) | 1.28 | | 1.13 | | 1.45 |
| **Cardiovascular Disease** | No | Reference | | | | |
|  | Yes | 1.11 | | 1.01 | | 1.22 |
| **Any Other Comorbidities** | None | Reference | | | | |
|  | Any | 1.29 | | 1.05 | | 1.59 |
| **Difficulty Performing at Least 1 Activity of Daily Living** | No | Reference | | | | |
|  | Yes | 1.07 | 0.96 | | 1.18 | |
| **Fall History in Past 12 Months** | No | Reference | | | | |
|  | Yes | 1.05 | | 0.94 | | 1.17 |
| **Radiation** | No | Reference | | | | |
|  | Yes | 1.06 | | 0.97 | | 1.17 |
| **Grade** | Grade I | Reference | | | | |
|  | Grade II | 0.96 | | 0.87 | | 1.07 |
|  | Grade III | 0.92 | | 0.81 | | 1.04 |
|  | Grade IV | 0.46 | | 0.08 | | 2.54 |
| **Age at Diagnosis** | Continuous | 0.99 | | 0.98 | | 1.00 |
| **Time from Survey to Diagnosis** | Continuous | 0.99 | | 0.98 | | 1.01 |
| **Sociodemographic Characteristics** | | | | | | |
| **Race/Ethnicity** | Non-Hispanic White | Reference | | | | |
|  | Non-Hispanic American Indian/Alaska Native/Asian or Pacific Islander | 1.20 | | 1.03 | | 1.39 |
|  | Non-Hispanic Black | 0.98 | | 0.83 | | 1.14 |
|  | Hispanic | 1.14 | | 0.98 | | 1.31 |
| **Marital Status** | Married | Reference | | | | |
|  | Divorced/Separated/Widowed | 1.03 | | 0.94 | | 1.14 |
|  | Never married | 0.78 | | 0.60 | | 1.02 |
| **Education** | College degree or more | Reference | | | | |
|  | High school graduate or GED | 0.89 | | 0.81 | | 0.99 |
|  | Less than high school | 0.77 | | 0.67 | | 0.88 |

BMI: body mass index; OR: odds ratio; CI: confidence interval; GED: general education development

*Represents a fully adjusted model; all variables in table were included in the model. The total number of breast cancer survivors included in this analysis is 1,551.

Supplemental Table 4. Modified Poisson Regression Results – Pulmonary Disease*

| **Variable** | **Category** | **Relative Risk** | | **95% Confidence Interval** | | |
| --- | --- | --- | --- | --- | --- | --- |
|  |  |  |  | **Lower** | | **Upper** |
| **Clinical Characteristics** | | | | | | |
| **BMI** | Normal (≥18.5-<25kg/m^2^) | Reference | | | | |
|  | Underweight (<18.5kg/m^2^) | 0.74 | | 0.54 | | 1.03 |
|  | Overweight (≥25-<30kg/m^2^) | 1.21 | | 1.07 | | 1.36 |
|  | Obese/Morbidly Obese (≥30kg/m^2^) | 1.25 | | 1.10 | | 1.41 |
| **Pulmonary Disease** | No | Reference | | | | |
|  | Yes | 1.01 | | 0.90 | | 1.14 |
| **Any Other Comorbidities** | None | Reference | | | | |
|  | Any | 1.35 | | 1.08 | | 1.68 |
| **Difficulty Performing at Least 1 Activity of Daily Living** | No | Reference | | | | |
|  | Yes | 1.10 | 0.99 | | 1.21 | |
| **Fall History in Past 12 Months** | No | Reference | | | | |
|  | Yes | 1.05 | | 0.94 | | 1.17 |
| **Radiation** | No | Reference | | | | |
|  | Yes | 1.07 | | 0.98 | | 1.18 |
| **Grade** | Grade I | Reference | | | | |
|  | Grade II | 0.96 | | 0.87 | | 1.07 |
|  | Grade III | 0.91 | | 0.81 | | 1.04 |
|  | Grade IV | 0.46 | | 0.08 | | 2.51 |
| **Age at Diagnosis** | Continuous | 0.99 | | 0.98 | | 1.00 |
| **Time from Survey to Diagnosis** | Continuous | 0.99 | | 0.97 | | 1.01 |
| **Sociodemographic Characteristics** | | | | | | |
| **Race/Ethnicity** | Non-Hispanic White | Reference | | | | |
|  | Non-Hispanic American Indian/Alaska Native/Asian or Pacific Islander | 1.18 | | 1.02 | | 1.36 |
|  | Non-Hispanic Black | 0.98 | | 0.84 | | 1.15 |
|  | Hispanic | 1.15 | | 0.99 | | 1.32 |
| **Marital Status** | Married | Reference | | | | |
|  | Divorced/Separated/Widowed | 1.02 | | 0.93 | | 1.12 |
|  | Never married | 0.79 | | 0.61 | | 1.02 |
| **Education** | College degree or more | Reference | | | | |
|  | High school graduate or GED | 0.89 | | 0.80 | | 0.98 |
|  | Less than high school | 0.77 | | 0.68 | | 0.89 |

BMI: body mass index; OR: odds ratio; CI: confidence interval; GED: general education development

*Represents a fully adjusted model; all variables in table were included in the model. The total number of breast cancer survivors included in this analysis is 1,576.

Supplemental Table 5. Modified Poisson Regression Results – Diabetes*

| **Variable** | **Category** | **Relative Risk** | | **95% Confidence Interval** | | |
| --- | --- | --- | --- | --- | --- | --- |
|  |  |  |  | **Lower** | | **Upper** |
| **Clinical Characteristics** | | | | | | |
| **BMI** | Normal (≥18.5-<25kg/m^2^) | Reference | | | | |
|  | Underweight (<18.5kg/m^2^) | 0.75 | | 0.54 | | 1.04 |
|  | Overweight (≥25-<30kg/m^2^) | 1.19 | | 1.06 | | 1.34 |
|  | Obese/Morbidly Obese (≥30kg/m^2^) | 1.22 | | 1.07 | | 1.38 |
| **Diabetes** | No | Reference | | | | |
|  | Yes | 1.15 | | 1.04 | | 1.27 |
| **Any Other Comorbidities** | None | Reference | | | | |
|  | Any | 1.26 | | 1.02 | | 1.56 |
| **Difficulty Performing at Least 1 Activity of Daily Living** | No | Reference | | | | |
|  | Yes | 1.08 | 0.98 | | 1.19 | |
| **Fall History in Past 12 Months** | No | Reference | | | | |
|  | Yes | 1.04 | | 0.94 | | 1.16 |
| **Radiation** | No | Reference | | | | |
|  | Yes | 1.07 | | 0.97 | | 1.18 |
| **Grade** | Grade I | Reference | | | | |
|  | Grade II | 0.96 | | 0.87 | | 1.07 |
|  | Grade III | 0.91 | | 0.80 | | 1.04 |
|  | Grade IV | 0.47 | | 0.09 | | 2.61 |
| **Age at Diagnosis** | Continuous | 0.99 | | 0.98 | | 1.00 |
| **Time from Survey to Diagnosis** | Continuous | 0.99 | | 0.98 | | 1.01 |
| **Sociodemographic Characteristics** | | | | | | |
| **Race/Ethnicity** | Non-Hispanic White | Reference | | | | |
|  | Non-Hispanic American Indian/Alaska Native/Asian or Pacific Islander | 1.16 | | 1.00 | | 1.34 |
|  | Non-Hispanic Black | 0.95 | | 0.81 | | 1.11 |
|  | Hispanic | 1.13 | | 0.98 | | 1.31 |
| **Marital Status** | Married | Reference | | | | |
|  | Divorced/Separated/Widowed | 1.01 | | 0.92 | | 1.11 |
|  | Never married | 0.78 | | 0.60 | | 1.01 |
| **Education** | College degree or more | Reference | | | | |
|  | High school graduate or GED | 0.88 | | 0.79 | | 0.97 |
|  | Less than high school | 0.76 | | 0.67 | | 0.87 |

BMI: body mass index; OR: odds ratio; CI: confidence interval; GED: general education development

*Represents a fully adjusted model; all variables in table were included in the model. The total number of breast cancer survivors included in this analysis is 1,579.

Supplemental Table 6. Modified Poisson Regression Results – Musculoskeletal Disease*

| **Variable** | **Category** | **Relative Risk** | | **95% Confidence Interval** | | |
| --- | --- | --- | --- | --- | --- | --- |
|  |  |  |  | **Lower** | | **Upper** |
| **Clinical Characteristics** | | | | | | |
| **BMI** | Normal (≥18.5-<25kg/m^2^) | Reference | | | | |
|  | Underweight (<18.5kg/m^2^) | 0.72 | | 0.52 | | 1.00 |
|  | Overweight (≥25-<30kg/m^2^) | 1.22 | | 1.08 | | 1.37 |
|  | Obese/Morbidly Obese (≥30kg/m^2^) | 1.24 | | 1.09 | | 1.40 |
| **Musculoskeletal Disease** | No | Reference | | | | |
|  | Yes | 1.22 | | 1.08 | | 1.37 |
| **Any Other Comorbidities** | None | Reference | | | | |
|  | Any | 1.13 | | 0.99 | | 1.28 |
| **Difficulty Performing at Least 1 Activity of Daily Living** | No | Reference | | | | |
|  | Yes | 1.06 | 0.95 | | 1.17 | |
| **Fall History in Past 12 Months** | No | Reference | | | | |
|  | Yes | 1.04 | | 0.93 | | 1.15 |
| **Radiation** | No | Reference | | | | |
|  | Yes | 1.07 | | 0.97 | | 1.18 |
| **Grade** | Grade I | Reference | | | | |
|  | Grade II | 0.97 | | 0.88 | | 1.08 |
|  | Grade III | 0.93 | | 0.82 | | 1.05 |
|  | Grade IV | 0.47 | | 0.09 | | 2.43 |
| **Age at Diagnosis** | Continuous | 0.99 | | 0.98 | | 1.00 |
| **Time from Survey to Diagnosis** | Continuous | 0.99 | | 0.98 | | 1.01 |
| **Sociodemographic Characteristics** | | | | | | |
| **Race/Ethnicity** | Non-Hispanic White | Reference | | | | |
|  | Non-Hispanic American Indian/Alaska Native/Asian or Pacific Islander | 1.18 | | 1.02 | | 1.37 |
|  | Non-Hispanic Black | 0.97 | | 0.83 | | 1.13 |
|  | Hispanic | 1.14 | | 0.99 | | 1.31 |
| **Marital Status** | Married | Reference | | | | |
|  | Divorced/Separated/Widowed | 1.01 | | 0.92 | | 1.11 |
|  | Never married | 0.80 | | 0.62 | | 1.03 |
| **Education** | College degree or more | Reference | | | | |
|  | High school graduate or GED | 0.89 | | 0.80 | | 0.99 |
|  | Less than high school | 0.76 | | 0.67 | | 0.87 |

BMI: body mass index; OR: odds ratio; CI: confidence interval; GED: general education development

*Represents a fully adjusted model; all variables in table were included in the model. The total number of breast cancer survivors included in this analysis is 1,572.
